# Supplementary material for: Activation-independent capture of free fatty acids at the bacterial cell envelope by BrtB
Source: mBio. 2026 Sep 9;17(10):e01132-26. doi: 10.1128/mbio.01132-26 (PMC13712857; doi:10.1128/mbio.01132-26)
Supplement: Supplementary file 1 [file mbio.01132-26-s0001.docx]

**Figure S1.** **Confirmation and quantification of ¹⁸O₂ labeling of hexanoic and dodecanoic acids by direct injection HRESIMS.** Stable isotope ¹⁸O₂ hexanoic acid and ¹⁸O₂ dodecanoic acid were generated by oxygen exchange in H₂¹⁸O under acidic conditions (Methods). Direct injection detected 3.47% and 0.91% of unlabeled hexanoic and dodecanoic acids respectively, together with mono¹⁸O (m/z +2.004) in 41.85% and 15.89% as well as di¹⁸O (m/z +4.008) isotopologues, confirming more than 95% of incorporation of one to two ¹⁸O atoms.

**Figure S2.** **Supplementation of *Synechocystis salina* LEGE 06099 (S06099) with ^18^O_2_-** **dodecanoic acid (C12) leads to the direct esterification of bartolosides into bartoloside fatty acid esters (B-FAs). (A)** Representation of the two different possible pathways taken by the supplemented ^18^O_2_-C12 fatty acid (FA): direct incorporation by BrtB would lead to the incorporation of two (monoester) or four (diester) ^18^O atoms; while prior activation of the FA by the acyl-acyl carrier protein synthetase (Aas) would lead to the incorporation of one (monoester) or two (diester) ^18^O atmos. **(B)** Schematic of the synthesis of ^18^O_2_ dodecanoic acid (C12) from H_2_^18^O and C12. **(C)** Structures of bartoloside A (**1**), mono- and didodecanoate esters (**9a**/**9b**, **10**) and their ^18^O-isotopologues generated upon supplementation of S06099 with ^18^O_2_-C12. **(D)** LC-HRESIMS-based detection of **1**, **9**, **10**, and ^18^O_2 or 4_-labeled **9** and **10** in ^18^O_2_-C12 supplemented cultures of S06099. Values next to each peak in extracted ion-chromatograms (EICs) correspond to peak height (ion counts). ACP, acyl–acyl carrier protein; Xyl, xylose.

**Figure S3. Supplementation of *Synechocystis salina* LEGE 06099 (S06099) with ^18^O_2_-hexanoic (C6) or with ^18^O_2_-dodecanoic (C12) acids leads to the direct esterification of bartolosides G into bartoloside fatty acid esters (B-FAs) G. (A)** Representation of the two different possible pathways taken by the supplemented ^18^O_2_-C12 fatty acid (FA): direct incorporation by BrtB would lead to the incorporation of two (monoester) ^18^O atoms, while prior activation of the FA by the acyl-acyl carrier protein synthetase (Aas) would lead to the incorporation of one (monoester) ^18^O atom. **(B)** Structures of bartoloside G (**4**), monohexanoate (**5**) monododecanoate (**11**) esters, and their ^18^O-isotopologues generated upon supplementation of S06099 with ^18^O_2_-C6 or ^18^O_2_-C12. **(C)** LC-HRESIMS-based detection of **4**, **5** and ^18^O_2_ labeled **5** in ^18^O_2_-C6 supplemented cultures of S06099. **(D)** LC-HRESIMS-based detection of **4**, **11** and ^18^O_2_ labeled **11** in ^18^O_2_-C12 supplemented cultures of S06099. Values next to each peak in extracted ion-chromatograms (EICs) correspond to peak height (ion counts); ACP, acyl–acyl carrier protein; Xyl, xylose.

**Source data S1 and S2 (excel). Extracted** **LC-MS peak area (area under the curve) of bartoloside-related metabolites, of phosphatidylglycerols (PGs) and sulfoquinovosyldiacylglycerols (SQDGs) after fatty acid (FA) supplementation.** S1, Samples 1–3, 5–7 and 9–11 were supplemented with 0.01, 0.05 and 0.5 mM of deuterated FA (*d*_15_C8 m/z + 15,09415 for one labeled chain and + 30.1883 for two labeled chain ; *d*_23_C12 m/z + 23.14426 for one labeled chain and m/z + 46.288 for two labeled chain) respectively, and samples 4, 8 and 12 received DMSO only. T1 samples were collected 24 h after supplementation and T2 samples were collected 7 days after supplementation. Di esters labeled species were detected with either one labeled FA chain (“*d*15, *d*23”) or two labeled FA chains (“*d*30, *d*46”). S2, Samples 1–3, 5–7 and 9–11 were supplemented with 0.01, 0.05 and 0.5 mM of deuterated FA (*d*_15_C8 m/z + 15,09415 for one labeled chain and + 30.1883 for two labeled chain ; *d*_23_C12 m/z + 23.14426 for one labeled chain and m/z + 46.288 for two labeled chain) respectively, and samples 4, 8 and 12 received DMSO only. T1 samples were collected 24 h after supplementation and T2 samples were collected 7 days after supplementation. PG- and SQDG-associated labeled species were search with either one labeled FA chain (“*d*15, *d*23”) or two labeled FA chains (“*d*30, *d*46”) and 0 to 4 unsaturations.

SourceData_S1_S2_C12_C8_supplementation_bartolosides_related_metabolites_and PGs_SQDGs.

**Figure S4.** ***Synechocystis salina* LEGE 06099 (S06099) bartoloside levels decrease with increasing supplementation of *d*_15_–octanoic acid (C8) while bartoloside fatty acid esters (B-FA) levels plateau. (A)** Design of labeled fatty acid (FA) dose-response experiment. Mid-exponential cultures (OD ~0.5) of S06099 were supplemented with 0.01, 0.05 or 0.5 mM *d*_15_–C8 (or DMSO as a control) and harvested after 24 h and 7 days for LC-HRESIMS analysis. **(B)** Sum of area under the curves (AUCs) for **1** and **4**. **(C)** Sum of AUCs for *d*-labeled octanoate esters (B-FAs; *d*_15_-**14**, *d*_30_-**15**). Bars show mean ± s.d. (n = 3 biological replicates); asterisks indicate significant differences between conditions (ns, not significant; * P < 0.05; ** P < 0.01; *** P < 0.001). All AUC values correspond to extracted ion chromatograms of the [M–H+HCOOH]^⁻^ formate adduct of the indicated species. S06099, *Synechocystis salina* LEGE 06099, AUC, area under the curve, DMSO, dimethyl sulfoxide; LC-MS, liquid chromatography-mass spectrometry.

**Figure S5.** **Bartoloside fatty acid esters (B-FAs) are hydrolyzed to hydroxybartolosides (B-OHs). (A)** Representation of proposed events following ^18^O_2_-dodecanoic acid (C12) supplementation: direct esterification by BrtB leading to the incorporation of two or four ^18^O atoms (two per each ester bond), followed by subsequent hydrolysis of the ester bonds to generate dihydroxylated bartolosides containing one or two ^18^O labeled B-OH. **(B)** Structures of B-OHs (**12**) and their ^18^O-isotopologues generated upon supplementation of *Synechocystis salina* LEGE 06099 (S06099) with ^18^O_2_-C12. **(C)** LC-HRESIMS-based detection of **12** and ^18^O_3_ labeled **12** in ^18^O_2_-C12 supplemented cultures of S06099. Values next to each peak in the extracted ion-chromatograms correspond to peak height (ion counts). Xyl, xylose.

**Figure S6.** **Fatty acid supplementation does not change non-labeled glycolipid levels.** Extracted LC-MS peak areas (AUC) of non-labeled phosphatidylglycerols (PG; A) and sulfoquinovosyldiacylglycerols (SQDG; B) in *Synechocystis salina* LEGE 06099 after supplementation with *d₂₃*–C12 (0.01, 0.05, or 0.5 mM; in DMSO) or DMSO alone. Samples were collected 24 h and 7 days after supplementation. Bars show mean ± s.d., with individual biological replicates overlaid (n = 3). AUC values correspond to extracted ion chromatograms of the [M–H+HCOOH]^⁻^ formate adducts. AUC, area under the curve; DMSO, dimethyl sulfoxide.

**Figure S7.** **Bartoloside fatty acid ester (B-FA) acyl composition is stable, while anionic glycerolipids remodel with temperature.** Sum of area under the curves (AUCs) for B-FAs (A), **phosphatidylglycerols (**PGs) (B) and **sulfoquinovosyldiacylglycerols (SQDGs) (C). Anionic glycerolipids** here refers to **PGs** and **SQDGs** quantified by negative-mode LC-MS; other lipid classes were not assayed. Sampling days were chosen to include one point near **OD ~0.5** at each temperature, to compare **matched physiological states**. “1”, “2”, “3” refers to the different triplicates in each condition. For each triplicate, the AUC per compound was extracted and then transformed into proportion. Asterisks indicate significant temperature effects (17°C vs 30°C) on replicates-level indices (B-FA/PG/SQDG long-chain content (top) and unsaturation content (bottom); Welch’s t-test with Benjamini–Hochberg correction, p < 0.05). “ns” marks non-significant contrasts and “***” marks p < 0.001.

**Source data S3. Extracted** **LC-MS peak areas (area under the curve) of bartolosides fatty acid esters, PGs and SQDGs observed at 17°C and 30°C in S06099.**

Source_Data_S3_temperature_AUC.

**Source data S4:** **Optical Density (OD) of *Synechocystis salina* LEGE 06099 (S06099) cultures used for the transcriptomics and metabolomics assay and extracted** **LC-MS peak areas (AUC) of bartoloside related metabolites.**

Source_data_S4_OD_metabolomics

**Figure S8.** **Hydroxy-bartoloside (B-OH) profiles grouped by type.** Total LC-MS peak area (AUC) of each B-OH type, obtained by summing separately the AUC of all detected species containing that hydroxyl group (**13a/b**, **12a/b**, **6**, **8**), at 30 min and 6 h in dodecanoic-acid-treated vs control cultures; bars show mean ± s.d. for biological replicates and asterisks indicate statistical significance (* P < 0.05; ** P < 0.01; ns, not significant). B-OH, hydroxy bartoloside; AUC values correspond to extracted ion chromatograms of the [M–H+HCOOH]^⁻^ formate adducts. AUC, area under the curve; DMSO, dimethyl sulfoxide.

**Figure supplement S9.** **Sample-wise Pearson correlation of RNA-seq libraries.** Heatmap showing pairwise Pearson correlation coefficients between all RNA-seq samples, calculated from normalized gene expression values. Samples cluster primarily by timepoint (T1, 30 min; T2, 6 h), with additional separation by treatment at 6 h, revealing near-identical expression profiles across all libraries (r > 0.99), and no sample deviated from this pattern. Color scale indicates the Pearson correlation coefficient (r); DMSO, dimethyl sulfoxide.

**Figure S10. Time-dependent gene expression in *Synechocystis salina* LEGE 06099 (S06099).** Volcano plot of differential expression for 6 h versus 30 min samples in S06099, testing the effect of time. Points show log_2_ fold change (6 h/30 min) versus –log_10_ adjusted p value (FDR); genes meeting |log_2_FC| ≥ 1 and FDR ≤ 0.05 are highlighted as significantly up- or downregulated.

**Table S1 (excel).** **S1,** **differentially expressed genes between 30 min and 6 h regardless of treatment. S2, Top 10 non-contaminant proteins per gel band piece (LC-MS/MS).** For each excised SDS–polyacrylamide gel band piece (F1–F4), the table lists the **ten most abundant protein entries** after excluding proteins flagged as **Contaminant** in the proteome export (Source data 3-6). Abundances correspond to Source data 3-6 **Abundances (Normalized)** for the indicated sample. “Rank (non-contam)” is the rank within that gel piece among non-contaminant proteins. “Coverage (%)” and “Peptides” report the sequence coverage and the number of identified peptides assigned to each protein, as reported in the Source data 3-6. Table_S1_S2.

**Text S1.** **BrtB amino acid sequence and annotated features.**

MANPFDVALTNLNSLYSGIPDVPVLQTYNSLNPYDISLTNLNNLSNFVIEPAVLPTAAGTYGPQISEIAFALGGGIITGALADLLSTELGIEIENEALITKFLSFWTPGSDYAFGQDYVEFIQGREGFDTIIGYDPGFNSALAPVQIDFVLGGPRQDFNFFAPLDEPARYVLGDWRTPYYVDNDIQSEGLNEFMYVSLFTLNPDEFSPDNAENHRIQLYGSLDNYYFVPTTVVIDGSALDPSLPSIPVPGTEIYYKTGQVFADLIAIVPFQDPNDPNLISAWIDFEGYLPPEQADLSGQTANPLLSGSPLLQLGYEGIDMGTATALAPDGDIYVAGSTSSLLGPFPRGGSDNFIARYNDDGSLEWIVQFGSTEFDIITDIVSDSAGNVYATGWTRGNVETGFGLAPGVQDNWVAKFDANGSQLWVEQFTIDGYLDRSMGIALDEVNGRIHLTGHSNTDGSVADTNDPAIVPNGNVVTWIAGFDANTGAEDYRNVFEVAQTSNSRGRIDEGFGIDVDDAGNVFSTGWAGENAYDVYLVKSDTDGAVLWTKSFGTGSNATQSYAWDVASDGTNAYILGWTQGELNTFRDQIPPTDEFAGNATAPLTPQNAYQGGTFDAFVAAYDTDGTELWTWHVGGTGDDGTFFGKIVAAGDFVYATGYTDGFIGTLGGSNAGDYDAWIGKFDKLTGQTAWIQQIGSTKLDYATGISVDGDDIFVTGFTEGSLGGLNGGASDAWVAKLNQDGALEAFNVSAPIAPPAPFAPPAPLTPPAPPAPDLSSLQSLGSTPQPTYPALGDSYGTNPFGTQPTQPTYDLSSMYQNAYYGGFGGYSGYGY

BrtB from S06099 is shown with three annotated regions: a glycine/aspartate-rich predicted calcium-binding motif consistent with RTX-like repeats (underlined grey), a proline-rich region proposed to disrupt β-sheet formation (blue), and the C-terminal glycine/serine-rich linker (green). BrtB is 88.6 kDa with a predicted pI of 3.73, contains ~12% glycine, and lacks cysteine residues.

**Source data S5**. Total exoproteome of *Synechocystis salina* LEGE06099 (S06099) without and with C16 supplementation.

**Source data S6-9 (excel).** **Proteome tables for gel band pieces F1-F4** (SourceData_S6_Fig7_F1; SourceData_S7_Fig7_F2 ; SourceData_S8_Fig7_F3 ; SourceData_S9_Fig7_F4).

**Figure S11.** **Electron microscopy of *Synechocystis salina* LEGE 06099 with and without FA supplementation.** Transmission electron micrographs of S06099 cells grown without (S06099) and with (S06099 + C16) C16 fatty acid supplementation, and of *Synechocystis* sp. PCC 6803 (S6803) grown without fatty acid supplementation, showing the S-layer (star), outer membrane (chevron) and the peptidoglycan (diamond). Vesicle-like protrusions (arrowheads) at the cell surface can be observed across conditions in S06099. Scale bars, 200 nm (top panels and bottom left) and 100 nm (bottom-right zoom).

**Note S1. Attempts to generate Δ*brtB* and Δ*aas* mutants in *Synechocystis salina* LEGE 06099.**

To directly test the role of BrtB in the cellular responses described in this study including FFA esterification, bartoloside remodeling, and envelope organization, we undertook sustained efforts over several years to generate a chromosomal *brtB* deletion mutant in S06099 (plasmid construction described in Methods). A Δ*brtB* strain would have allowed unambiguous attribution of the observed esterification activity to BrtB, and provided a genetically defined background for transcriptomic, proteomic, and ultrastructural analyses. In parallel, we attempted to generate a Δ*aas* mutant, which would have been informative both as a transformation control and as an independent tool to determine whether exogenous FFAs that bypass BrtB are routed through Aas for canonical activation a key open question that our current data cannot resolve.

**DNA transfer attempts.** Natural transformation was performed across more than ten independent experiments, systematically varying: cell density at time of transformation (OD_730_ ~0.5–0.67); plasmid amount (2.7–10 µg); cell washing status (washed vs. unwashed with autoclaved distilled water); plating format (direct on agar vs. on nitrocellulose membranes); salt concentration (standard 25 g L⁻¹ vs. low-salt 2.5 g L⁻¹ vs. 5 g L⁻¹); phosphate regime (standard Z8 vs. low-phosphate medium following ^1^); recovery temperature (23 °C, 25 °C, 30 °C); and initial antibiotic concentration (10, 15, 30 µg mL⁻¹ kanamycin or neomycin).

Electroporation was also attempted as an alternative method of DNA delivery. For the *aas* knockout, a non-replicative knockout plasmid was assembled in pBluescript II using upstream (A1) and downstream (A2) homology arms amplified from S06099 gDNA (766 and 608 base pair, respectively). The arms were amplified with Phusion polymerase in GC buffer supplemented with DMSO (annealing 61 °C) using primers: A1_Forward (ATTATACTCGAGTAGGTAACTGTCAGTATGCTC); A1_Reverse (TTTATACTGCAGGTAAGCCAGCGGCAAAG), which introduce restriction sites XhoI/PstI; and A2_Forward (TTATCACTGCAGTCGACATTGGGCTCCG); A2_Reverse (TAATTAGGATCCACGGGAAAGGAAGTGAGGG), which introduce restriction sites PstI/BamHI. The kanamycin resistance cassette was inserted between the arms following the same cloning strategy as described for the *brtB* knockout plasmid (Methods). Transformation of the *aas* knockout construct was performed under identical conditions to those described above for *brtB*.

Conjugation using the replicative vector pEF07 in a triparental mating format (described in Methods) was also attempted to test an alternative DNA delivery route.

**Attempts in additional strains.** To determine whether the transformation barrier was specific to S06099, conjugation using the replicative vector pEF07 (pSEVA251::sfGFP; RSF1010 origin; Ptrc.x.lacO promoter) was additionally attempted in close relatives bartolosides producers, namely *Synechocystis salina* LEGE 00031 and 00041 ^2^, following the triparental mating protocol described in Methods. No stable GFP-expressing exconjugants were recovered from either strain after two weeks of incubation and monitoring.

**Antibiotic tolerance and failed segregation.** Colonies tolerant to kanamycin concentrations up to 75 µg mL⁻¹ on solid medium were recovered after extended incubation. However, exposure to 100 µg mL⁻¹ kanamycin was uniformly lethal to all candidates, and critically, no putative transformant survived selective liquid culture at 50 µg mL⁻¹. Colony PCR from plates at 30–50 µg mL⁻¹ consistently yielded amplicons for both the kanamycin resistance cassette and the wild-type *brtB* locus simultaneously, indicating that putative transformants harbored both the endogenous gene and the resistance cassette rather than a true gene replacement. This pattern was reproducible across more than 40 individually screened colonies. Stepwise increases in kanamycin concentration over multiple transfer rounds failed to drive full segregation to a *brtB*-null genotype. Genomic DNA extracted from the most promising candidates and subjected to PCR with flanking primers always revealed the wild-type *brtB* allele and no kanamycin cassette. An identical outcome was obtained for all *aas* KO candidates: co-detection of kanamycin cassette and wild-type *aas* amplicons in colony PCR, marginal survival on selective solid medium, and failure to proliferate in selective liquid culture.

The parallel failure of brtB and aas knockouts, combined with the polyploid nature typical of cyanobacteria, suggests that the primary obstacle is incomplete chromosomal segregation rather than gene essentiality per se. The organism maintains multiple chromosome copies, and selection pressure may be insufficient to eliminate all wild-type copies, particularly when the target gene confers a fitness advantage or when DNA uptake efficiency is low. An alternative interpretation is that the kanamycin-tolerant colonies recovered on selective plates were not true transformants but rather spontaneous kanamycin or neomycin resistant contaminants possibly arising from other bacteria present in the culture environment which would account for both their survival on selective medium and the consistent absence of the kanamycin cassette from genomic DNA extracts. Distinguishing between these scenarios was not possible without stable transformants to analyze further. An additional, speculative possibility is that the bartoloside-rich envelope of S06099 presents a physicochemical barrier to DNA entry, consistent with the proposed role of these lipids in membrane organization and with the observation that strains harboring the BrtB system cluster phylogenetically in a group that has so far proven refractory to stable transformation in our hands. Direct experimental discrimination between these hypotheses would require genetic and cell biological tools not currently available for this organism.

**References**

1.	Pope, M. A., Hodge, J. A., & Nixon, P. J. (2020). *An Improved Natural Transformation Protocol for the Cyanobacterium Synechocystis sp. PCC 6803*. https://doi.org/10.3389/fpls.2020.00372

2.	Reis, J. P. A., Freitas, S., Procházková, T., & Leão, P. N. (2024). Expanding the Diversity of the Cyanobacterial Dialkylresorcinol Bartoloside Family. *Journal of Natural Products*. https://doi.org/10.1021/acs.jnatprod.4c00832
